# The Development, Fine Specificity, and Importance of High-Avidity Antibodies to VAR2CSA in Pregnant Cameroonian Women Living in Yaoundé, an Urban City

**DOI:** 10.3389/fimmu.2021.610108

**Published:** 2021-02-26

**Authors:** Koko Vanda, Naveen Bobbili, Masako Matsunaga, John J. Chen, Ali Salanti, Rose F. G. Leke, Diane Wallace Taylor

**Affiliations:** ^1^ Department of Tropical Medicine, Medical Microbiology and Pharmacology, John A. Burns School of Medicine, University of Hawaii, Honolulu, HI, United States; ^2^ Department of Quantitative Health Sciences, John A. Burns School of Medicine, University of Hawaii, Honolulu, HI, United States; ^3^ Centre for Medical Parasitology, Department for Immunology and Microbiology, Faculty of Health and Medical Sciences, University of Copenhagen and Department of Infectious Disease, Copenhagen University Hospital, Copenhagen, Denmark; ^4^ Faculty of Medicine and Biomedical Research, The Biotechnology Center, University of Yaoundé 1, Yaoundé, Cameroon

**Keywords:** *Plasmodium falciparum*, pregnant women, placental malaria, VAR2CSA, antibody avidity, urban cities

## Abstract

Pregnant women infected with *Plasmodium falciparum* often produce antibodies (**Abs**) to VAR2CSA, a ligand that binds to placental chondroitin sulfate A causing placental malaria (**PM**). Antibodies to VAR2CSA are associated with improved pregnancy outcomes. Antibody avidity is a surrogate marker for the extent of maturation of the humoral immune response. Little is known about high avidity Abs to VAR2CSA for women living in urban African cities. Therefore, this study sought to determine: i) if high avidity Abs to full-length VAR2CSA (**FV2**) increase with gravidity in women in Yaoundé, Cameroon exposed to ~ 0.3-1.1 infectious mosquito bites per month, ii) if high avidity Abs to FV2 are directed against a specific region of VAR2CSA, and iii) if having high avidity Abs to FV2 improve pregnancy outcomes. Plasma samples collected at delivery from 695 women who had Abs to FV2 were evaluated. Ab levels and the Avidity Index (**AI**), defined as the percent Abs remaining bound to FV2 after incubation with 3M NH_4_SCN, were determined. Similar Ab levels to FV2 were present in women of all gravidities (G1 through 6+; *p*=0.80), except significantly lower levels were detected in PM−negative (PM−) primigravidae (*p <*0.001). Median Ab avidities increased between gravidity 1 and 2 (*p*<0.001) and remained stable thereafter (G3-G6+: *p*=0.51). These results suggest that B cell clonal expansion began during the first pregnancy, with clonal selection primarily occurring during the second. However, the majority of women (84%) had AI <35, a level of high avidity Abs previously reported to be associated with improved pregnancy outcomes. When plasma from 107 Cameroonian women was tested against 8 different regions of FV2, high avidity Abs were predominately restricted to DBL5 with median AI of 50 compared to AI <25 for the other domains. The only significance influence of high avidity Abs on pregnancy outcome was that babies born to mothers with AI above the median were 104 g heavier than babies born to women with AI below the median (*p*=0.045). These results suggest that a vaccine that boosts maturation of the immune response to VAR2CSA may be beneficial for women residing in urban areas.

## Introduction

*Plasmodium falciparum* infections during pregnancy may be harmful to both the mother and the developing fetus. Malaria-infected erythrocytes (**IE**) sequester in the intervillous space (**IVS**) of the placenta and stimulate an inflammatory response, thereby increasing the risk of maternal complications, stillbirths, premature deliveries, reduced infant birthweights, and low birthweight babies (1–4). Primigravidae are more susceptible to the effects of placental malaria (**PM**) than multigravida women. Sequestration is mediated primarily by an antigen expressed on the surface of IE called VAR2CSA that binds to chondroitin sulfate A (CSA) on syncytial trophoblasts lining the IVS (5, 6). Since CSA is only expressed in the placenta, IE expressing VAR2CSA are normally quickly eliminated in children and non-pregnant adults before an immune response is induced. However, when women are infected with *P. falciparum* for the first-time during pregnancy, they are exposed to VAR2CSA and a primary immune response is induced. The presence of antibodies (Abs) to VAR2CSA, especially in subsequent pregnancies, reduces the severity of PM and has been associated with increased infant birthweight, longer periods of pregnancy, reduced prevalence of PM, and reduced risk of low birthweight (LBW) babies (6–10) [reviewed in (11)]. Thus, immunity to PM and VAR2CSA is unique, in that it is pregnancy-associated and gravidity dependent.

Most studies assessing the benefits of Abs to VAR2CSA have been conducted in areas with high malaria transmission. The impact of Abs to VAR2CSA on pregnancy outcomes in urban settings with intermediate or low levels of transmission is less clear, in part, because a large sample size is needed to detect small changes. Currently, neither the amount, specificity nor immunological properties of Abs to VAR2CSA needed to improve pregnancy outcomes is known. In general, high Ab levels to VAR2CSA are associated with infection, not protection (11). Thus, many questions remain about the natural acquisition of Abs to VAR2CSA in pregnant women and their role in pregnancy outcomes in different transmission settings.

Antibody avidity, or functional affinity, is often listed as a functional assay, in that it measures the overall strength of binding of Abs circulating in plasma to an antigen (12). During affinity maturation, antigen is presented on follicular dendritic cells within germinal centers and B cells with B-cell receptors (sIg) that can outcompete circulating Abs and other maturing B cells for the antigen will be selected; thereby, increasing Ab avidity. Thus, Ab avidity is a marker for the extent of maturation of the humoral response against an antigen. Several studies have suggested that high avidity Abs to VAR2CSA might be beneficial to pregnant women residing in areas with intense malaria transmission. For example, pregnant women with high levels of high avidity Abs to VAR2CSA (i.e., >35% of Ab remained bound after treatment with 3M NH_4_SCN) early in pregnancy, had a reduced risk of placental malaria at delivery; whereas, having high Ab levels to VAR2CSA *per* se was not associated with improved pregnancy outcome (13). A second study, designed to identify differences in the fine-specificity of Abs to VAR2CSA between PM -positive (**PM+**) and PM− multigravid women, found that the only significant difference was the presence of higher levels of high avidity Abs to full-length VAR2CSA (**FV2**) among the 21 assays studied using VAR2CSA-associated recombinant proteins (14). It remains unclear, however, if Ab avidity plays a direct role in enhancing inhibition or releasing IE bound to trophoblasts or if avidity is simply a marker of maturation of the overall immune response to VAR2CSA.

The role of Abs to VAR2CA in a low to intermediate transmission areas, such as the city of Yaoundé, Cameroon, where pregnant women were exposed to ~0.3 to 1.1 infectious mosquito bites per month, remains unclear (15, 16). Tutterrow et al., followed 50 women throughout pregnancy who were positive for *P. falciparum* (Pf) parasites either by microscopy or PCR early in the second trimester and reported that 60% of the women who were PM− at delivery, lacked Abs to FV2 (17). These data suggest that Abs to antigens other than VAR2CSA assist in clearing infected erythrocytes. Several studies have shown that Abs to other non-VAR2CSA *P. falciparum* antigens were associated with improved pregnancy outcomes in women residing in Yaoundé, Cameroon (8, 18). Thus, the prevalence, amount and importance of high avidity Abs to VAR2CSA in areas with relatively low malaria transmission remains to be determined.

The purpose of the current study was to determine if: i) high avidity Abs to full-length VAR2CSA (**FV2**) increased with gravidity in women in Yaoundé, Cameroon prior to implementation of intermittent preventive treatment (**IPTp)**, ii) high avidity Abs to FV2 were directed against a specific region of FV2, and iii) having high avidity Abs to FV2 at delivery was associated with reduced prevalence of PM or improved infant birthweight.

## Materials and Methods

### Ethical Approvals

The original study, conducted between 1995 and 2001, was approved by the National Ethics Committee, Cameroon and the IRB, Georgetown University (IRB#:1994-158) for collection of plasma, placental tissue and clinical information. Women participating in the study gave written informed consent. The use of the de-identified, archival plasma samples and clinical information in the current study were determined to be exempt from human subjects’ research by the Committee on Human Subjects, University of Hawaii-Manoa (CHS#21891).

### The Study Design

#### Clinical and Laboratory Data

Plasma used in this study were consecutively collected at delivery between 1995 and 2001 in Yaoundé, Cameroon, at the Biyem Assi District Hospital where care was provided to women living in the adjacent area and at the Central Maternity Hospital, a referral hospital for a diverse group of women (19). At the time of recruitment, information on the woman’s pregnancy and malaria histories were recorded on a standardized questionnaire, a heparinized peripheral blood sample was drawn, and a biopsy of the placenta was excised. In the laboratory, thick and thin slides were prepared of maternal peripheral and placental IVS blood and impression smears were made using the excised placental tissue for detection of *P. falciparum* infected erythrocytes (**IE**). Histological sections of the placenta were also prepared to confirm infection. A woman was considered to be PM+ if parasites were detected in either the placental blood, impression smears or by histology. Maternal anaemia was assessed by determining the packed cell volume (**PCV**) (19). All data were maintained in a password-protected archival database. The deidentified plasma samples were stored at −80°C until used, except during transportation on dry ice from the Biotechnology Center, Cameroon to Georgetown University and later from the Georgetown University to the University of Hawaii. A total of 1,649 plasma samples and corresponding clinical information were available. An advantage of using the clinical data and plasma from this group is that the women were recruited before the implementation in 2004 of IPT; therefore, the women developed natural immunity to PM that aided in clearing their placental infections. Post-IPTp, placental parasitemia were either prevented or eliminated by sulfadoxine–pyrimethamine treatment, making it impossible to assess the role of Abs to VAR2CSA in naturally-acquired immunity. HIV status of the women was not determined, but the prevalence in pregnant women attending urban antenatal clinics at the time of sample collection was 7.1% (20).

#### Sample Selection

The current study on Ab avidity is an extension of a study conducted in 2017 that used data from the archival samples in statistical predictive models to identify the best combination of antigens associated with absence of PM (18). The study design, sample selection, and results of that study have been reported previously (8, 18). In the 2017 study, plasma samples from 341 PM+ women who delivered live babies ≥28 weeks of gestation, along with three times the number (n=1,036) of randomly-selected PM−negative (PM−) women were screened at a 1:200 dilution in a bead-based multiplex assay for Abs to 17 VAR2CSA-associated antigens (FV2, DBL 1-6 of the FCR3, 3D7 and 7G8 lines, ID1-ID2a (FCR3 and 3D7) and 11 antigens reported to be associated with immunity to *P. falciparum* (AMA-1, CSP, EBA-175, LSA1, MSP1, MSP2, MSP3, MSP11, Pf41, Pf70 and RESA) (18). In addition, plasma from 30 males of equivalent age residing in Yaoundé served as negative controls for the var antigens. The resulting database, consisting of over 74,000 Ab data points, allowed us to evaluate each sample for potential Ab deterioration due to long-term storage and to identify the samples with Abs to FV2. The cut-off for Ab-positivity was the mean + 2SD of the male controls. A total of 695 women were identified who were Ab+ for FV2. Thus, plasma and clinical information from the 695 Ab+ women, and plasma from 20 age-matched sympatric males, were evaluated in the avidity assay.

#### The Multiplex Assay for Measuring Ab Levels and Avidity to FV2

The multiplex-immunoassay for measuring Abs to FV2 and the avidity assay were detailed previously (13, 14). In brief, FV2 of the FCR3 line was produced in baculovirus as previously described (21). The antigen was covalently coupled at saturating concentrations to SeroMap beads (Luminex) overnight according to the manufacturer’s directions. Beads were then blocked with PBS+1% bovine serum albumin (BSA) and resuspended to 2,000 beads/50µl. Ab levels and avidity were determined by combining 50µl of plasma diluted 1:300 in PBS+1% BSA (pH 7.2) with 50µl of coupled beads (total dilution of plasma =1:600) in duplicate in filter microtitre wells. Following incubation at room temperature (**RT**) on a rotary shaker for 1 hr., beads were washed twice with PBS+0.05% Tween 20 and once with 1% BSA in PBS. Then, one of the paired wells was incubated with 100µl of PBS and one well with 100µl of 3M NH_4_SCN for 30 min are RT. After washing, beads were incubated with 100µl of secondary antibody (R-phycoerythrin-conjugated, Affinity Pure F(ab’)_2_ fragment, Goat anti-human IgG Fc fragment specific, Jackson Immunoresearch, West Grove, PA) diluted to 2µg/ml in PBS-1% BSA was added to each well and incubated in the dark on a rotary shaker for 1 hr. Beads were washed, re-suspended in 100µl PBS-1% BSA and 85µl of the microsphere suspension was analyzed using a Liquichip M100 reader (Qiagen, Valencia, CA). The reader was programmed to read a minimum of 100 beads per spectral address, DD Gate 7,500–15,000. Results for Ab levels were expressed as median fluorescence intensity (**MFI**), where the linear part of the MFI binding curve extended from ~1,000 to >25,000 MFI (r coefficient of >0.90 for Ab dilution versus MFI) (22). Results from the avidity assay are expressed as an avidity index (**AI**) that equals the percentage of Abs that remains bound to FV2 after treatment with the chaotrope, using the formula (AI = [MFI beads with salt]/[MFI beads with no salt] x100). The entire assay was repeated by two investigators (KV, NB). When inadequate bead counts were obtained, the samples were re-run. The MFI for the two replicates were averaged and used for data analysis. Samples with MFI greater than the mean + 2 SD for the negative male controls were considered to be Ab+.

#### Measuring Ab Avidity to Different VAR2CSA Domains

The following Duffy-binding like (**DBL**) domains of VAR2CSA were coupled to SeroMap beads as described above: DBL1 (3D7), and the FCR3 lines of 1D1-2a, DBL 1 + 2, DBL2, DBL3, DBL4, DBL5, DBL6, and FV2. Characteristics of the recombinant proteins have been detailed previously (23, 24). The recombinant proteins were covalently coupled at saturating amounts to beads with different spectral addresses and then pooled to create a 10-plex. In establishing the assay, results from the 10-plex were compared with each of the antigens as a mono-plex to confirm that competition among the antigens did not occur. For the assay, 50µl of plasma diluted 1:300 was combined with 50µl of the 10-plex (2,000 beads of each antigen) for 60 min at RT. The remainder of the methods was the same as that described above. A total of 107 plasma samples with known MFI to FV2 were screened, including 20 sample with MFI of 5,000–10,000; 30 samples with 10,000–15,000; 28 samples with 15,000–20,000; and 29 samples with 20,000–25,000 MFI.

### Statistical Analysis

Demographic, clinical, and parasitological characteristics were first summarized by descriptive statistics: frequencies and percentages for categorical variables, i.e., maternal anaemia status; means with standard deviations (SD) or medians with interquartile ranges (IQR) and 25^th^ and 75^th^ percentiles for continuous variables based on data distribution. To compare the groups (e.g., by PM status or AI status), t-tests (or Wilcoxon-Rank-Sum tests) and analysis of variance [ANOVA or Kruskal-Wallis tests (**KW**)] were used for continuous variables depending on distribution and number of the groups. Chi Square tests were used for categorical variables. Post-hoc comparison test was used, when overall significance was identified, to examine the difference between two specific gravidity groups or AI groups. The relationships between birthweight and AI, and between PM and AI after adjusting for age and gravidity were examined by multivariable linear and logistic regression models, respectively. The relationship between MFI and AI was also assessed using linear regression. Statistical significance was determined by *p*<0.05.

## Results

### Description of the Study Population

Among the 695 women studied, 460 women were PM− and 235 were PM+ (**Table 1**). PM+ women were younger (24.4 vs 26.3 years; p<0.001); had fewer pregnancies (2 vs. 3; p<0.001); had lower hematocrits (31.3% vs 33.9% PCV; p<0.001); and were more likely to be anemic (28.7% vs. 18.9%; *p* = 0.011) than PM− women. A minor difference in length of gestation was found between PM+ and PM− pregnancies (39.3 vs. 40 weeks, *p*=0.019), but no differences were evident in percentage of premature deliveries or proportion of LBW babies (**Table 1**). However, singleton babies born to PM+ mothers were an estimated 138 gm lower in birthweight and singletons born full-term were 141 gm lighter (*p* = 0.007, *p* = 0.002, respectively). Thus, among women who had Abs to FV2, mothers with PM+ appeared to have lower birthweight babies than those without PM.

**Table 1:** Characteristics of women with antibodies to full-length VAR2CSA.

	PM− n = 460	PM+ n = 235	*P* value^a^
Age, Years [mean ± SD]	26.3 ± 5.6	24.4 ± 5.5	<0.001
Gravidity [median (IQR)]	3 (2,5)	2 (1,4)	<0.001
Placental parasitemia % [median (IQR)]	0	0.68 (0.16, 2.5)	NS
Maternal hematocrit, % PCV [mean ± SD]^b^	33.9 ± 5.9	31.3 ± 5.6	<0.001
Maternal anemia status^c^ [%]	18.9	28.7	0.011
Weeks of Gestation [median (IQR)]^d^	40.0 (38.0, 41.0)	39.3 (38.0, 40.6)	0.019
Preterm deliveries [%]^d,e^	19.9% (83/418)	21.6% (42/204)	0.915
Infant birth weight, g [mean ± SD]^d^	3,131 ± 627	2,993 ± 600	0.007
Infant birthweight from term deliveries [mean ± SD]^d^	3,297 ± 459	3,156 ± 482	0.002
Low-birthweight [%]^d^	13.9	18.1	0.203

^a^Statistical Tests: t-tests for age, maternal hematocrit, and infant birthweight; Wilcoxon-Rank-Sum tests for gravidity and weeks of gestation; Chi square tests of independence for anaemia, preterm delivery, and low birthweight.

^b^Analysis was performed on 569 subjects for whom data were available.

^c^Women with hematocrits <30% were defined as anemic.

^d^Twins were excluded from the analysis: n=622 women for weeks of gestation, preterm deliveries; n=648 for infant birthweight and low birthweight; n=493 for infant birthweight of full-term deliveries.

^e^Preterm delivery was defined as <37 weeks of delivery.

### Antibody Levels to FV2 Were Lower in PM− Primigravidae, but Similar Among Other Women

Plasma samples collected at delivery from the 695 pregnant women and 20 age-equivalent males were screened for Abs to FV2 at a 1:300 dilution in the avidity assay. Overall, 644/695 (93%) women had MFI to FV2 greater than the mean+2 SD of the male controls; whereas, 51 women, who originally tested positive at a 1:200 dilution, had MFI below the cut-off at a 1:300 dilution. Thus, results from only 644 women were included in the analysis.

**Figure 1:**
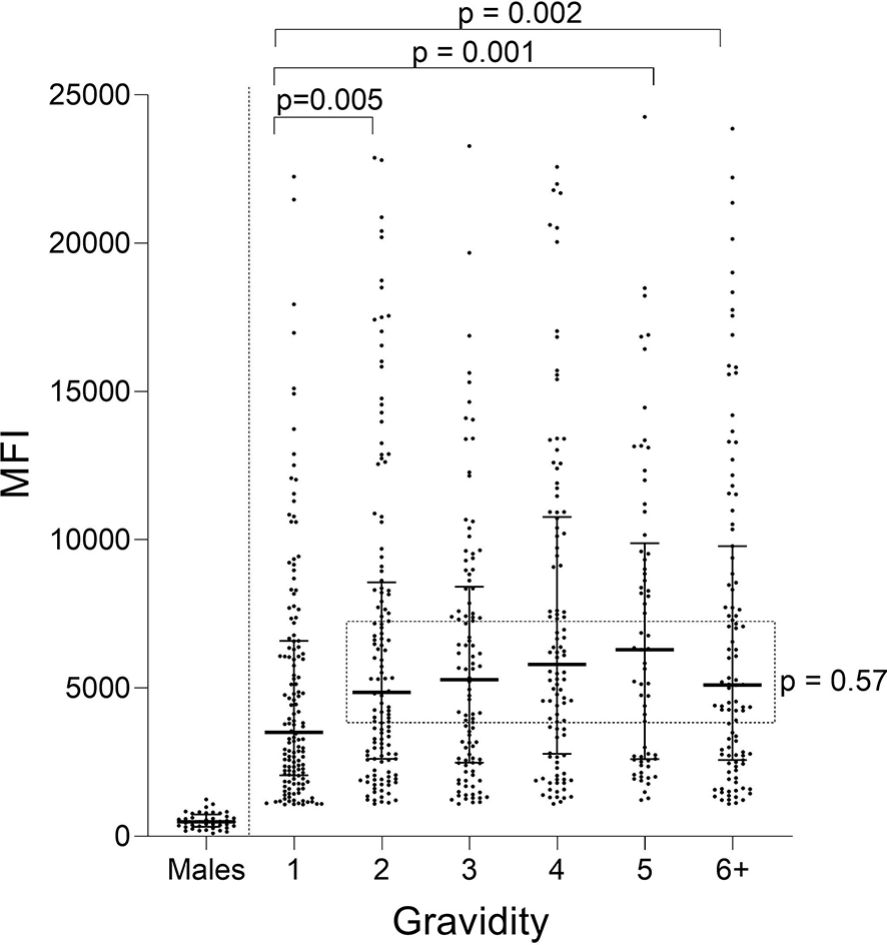
Antibody levels to full-length FV2 at delivery in different gravidity groups. A significant difference in median Ab levels was detected among the six gravidity (G) groups (Kruskal-Wallis test: *p* = 0.002). Post hoc pairwise comparison showed that primigravidae (G1) had significantly lower Ab levels to FV2 than each of the other gravidity groups. Ab levels among groups shown in the rectangle did not differ significantly (Kruskal-Wallis test: *p* = 0.57). n = 644 women and 20 males (replicates shown, n=40 data points). Horizontal lines represent medians and IQR.

Ab levels to FV2 for women with different gravidity (G1 through ≥G6) were compared (**Figure 1**). A difference in median Ab levels was found among the groups (Kruskal-Wallis test (**KW**): *p* = 0.002) with an apparent up-ward trend from primigravidae (G1) through grand-multigravid mothers (≥G6) (**Figure 1**). However, *post hoc* pairwise comparisons showed that the only significant difference in Ab levels among the groups was between G1 and G2 (*p* = 0.005). There was no difference in Ab levels between G2 and G6+ women (KW: *p* = 0.57: dotted rectangle in **Figure 1**).

**Figure 2:**
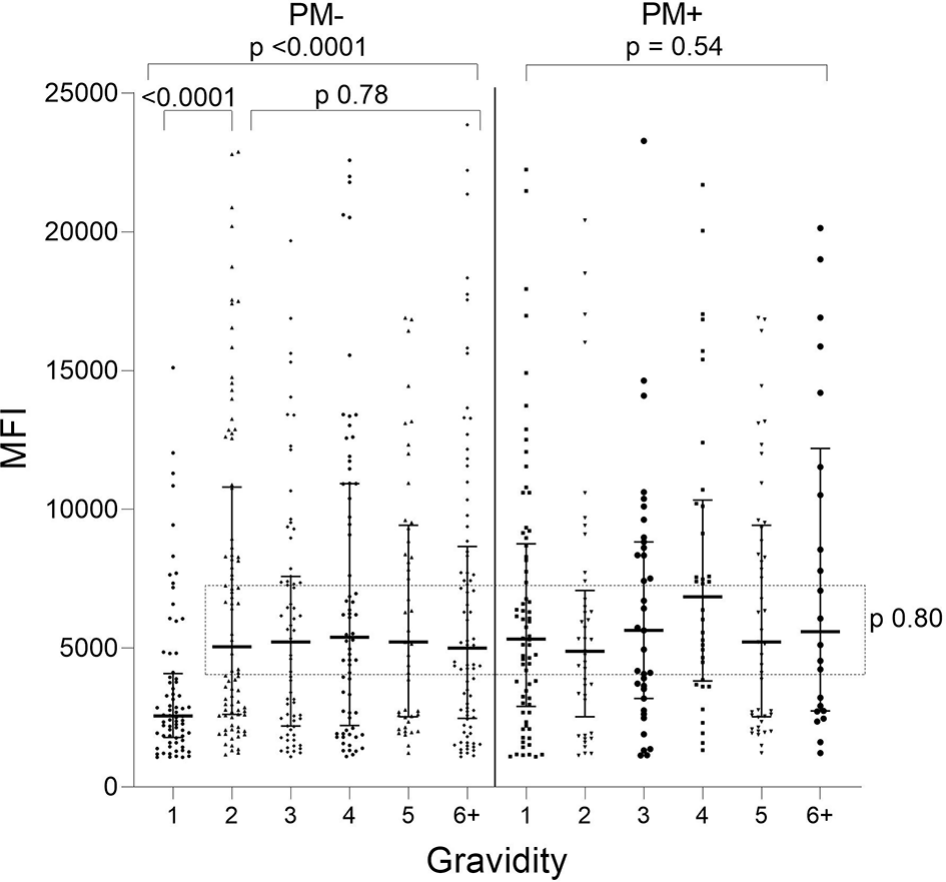
Antibodies to FV2 at delivery in PM− and PM+ women. Antibody levels were significantly lower in PM− primigravidae (G1) than G2 (Mann-Whitney test: *p <*0.001), but no significant difference was found among the gravidity groups shown in the rectangle (Kruskal-Wallis test: *p* = 0.80). n = 644 women. Horizontal lines represent medians and IQR.

When Ab levels were evaluated based on presence/absence of PM, only PM− primigravidae had significantly lower Ab levels than women in the other groups (e.g., primigravidae PM− vs PM+ p<0.001; PM− primi- vs PM− secondi-gravidae; *p*<0.001) (**Figure 2**). No difference in Ab levels was found among the other PM− gravidity groups (KW: *p* = 0.78) or among PM+ women G1 though G6+ (KW: *p* = 0.54). Thus, the distribution of Ab levels to VAR2CSA was similar among all the gravidity groups (*p* = 0.80), except for lower Ab levels in first-time mothers who were PM− at delivery.

### Antibody Avidity to FV2 Increased Between Gravidity 1 and Gravidity 2

**Figure 3:**
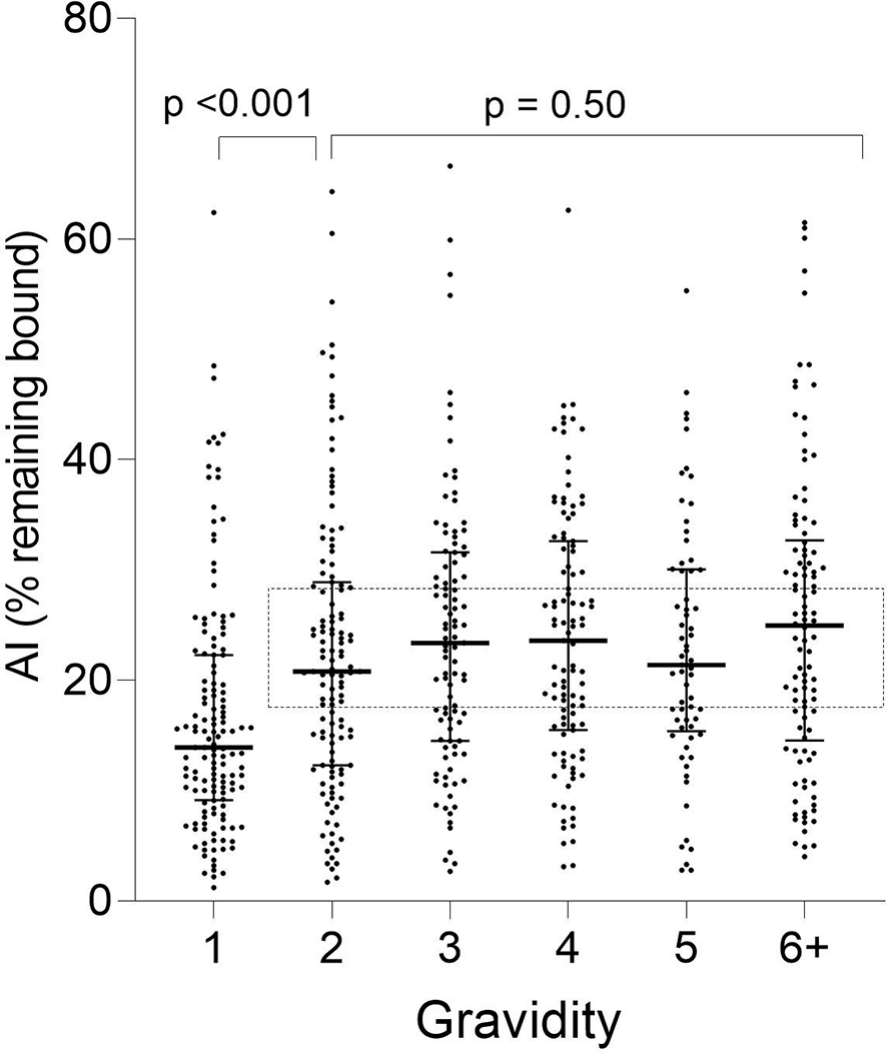
Distribution of Ab avidity (AI) among pregnant women at delivery. AI were significantly lower in G1 than G2 (*p <*0.001), but AI did not change thereafter (rectangle: Kruskal-Wallis test: G2 through G6+ *p* = 0.50). Horizontal lines represent medians and IQR.

A significant increase in median AI occurred between G1 and G2 (*p* < 0.001) and then AI leveled off thereafter (KW test: *p* = 0.50) (**Figure 3**). Thus, the predominant increase in AI occurred between women in their first and second pregnancies.

**Figure 4:**
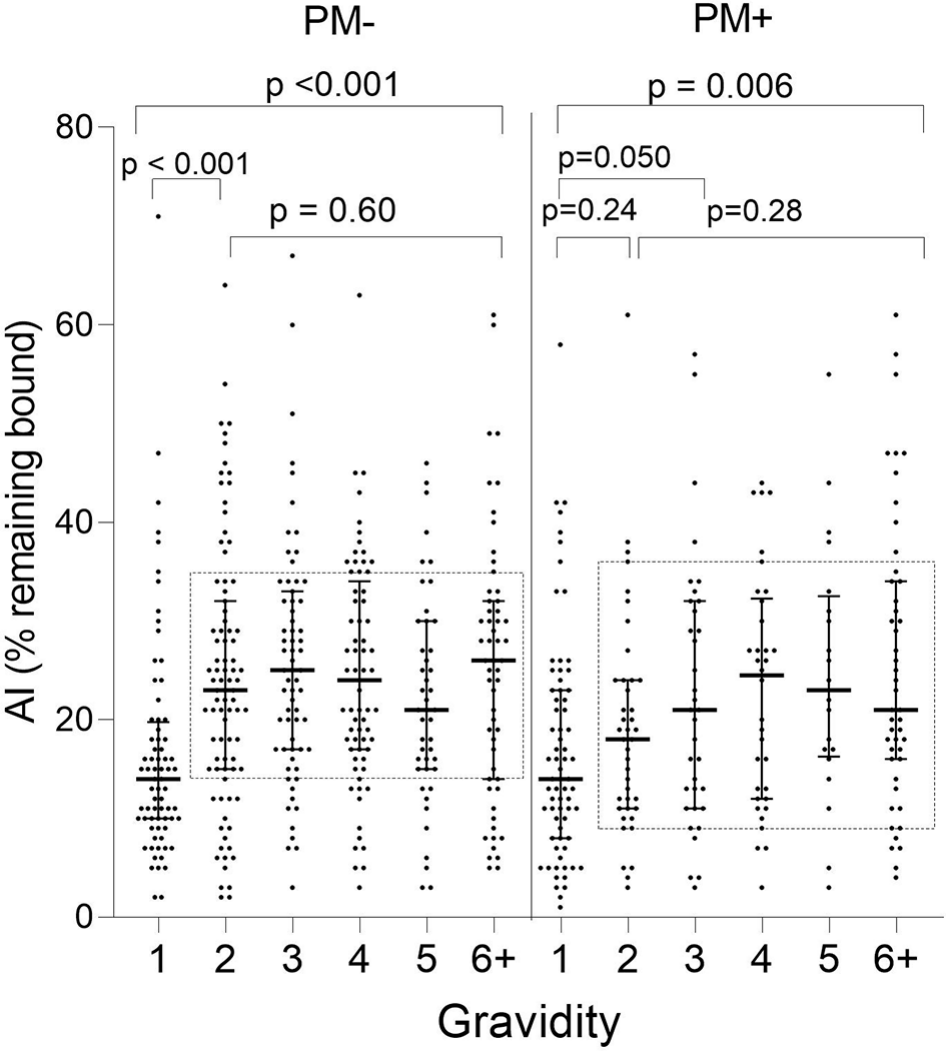
Comparison of Ab avidity (AI) at delivery in PM− and PM+ women. A significant increase in AI occurred in PM− women between G1 and G2 women (*p* < 0.001) and between G1 and G3 in PM+ women (*p* = 0.05), but AI did not increase significantly thereafter (samples within rectangles for G2 through G6+: Kruskal-Wallis test: PM− *p* = 0.60; PM+ *p* = 0.28). Horizontal lines represent medians and IQR.

When stratified by placental-malaria status at delivery, AI values were significantly lower in PM− primigravidae than secundigravidae (*p* < 0.001) and lower in PM+ primigravidae than G3 women (*p* = 0.050) (**Figure 4**). Thereafter, the AI leveled off (KW test: PM− *p* = 0.60; PM+ *p* = 0.28). Thus, median AI values were lower in both PM− and PM+ primigravidae, but significantly increased during the second pregnancy in PM− mothers and between the first and third pregnancy in PM+ women.

**Figure 5:**
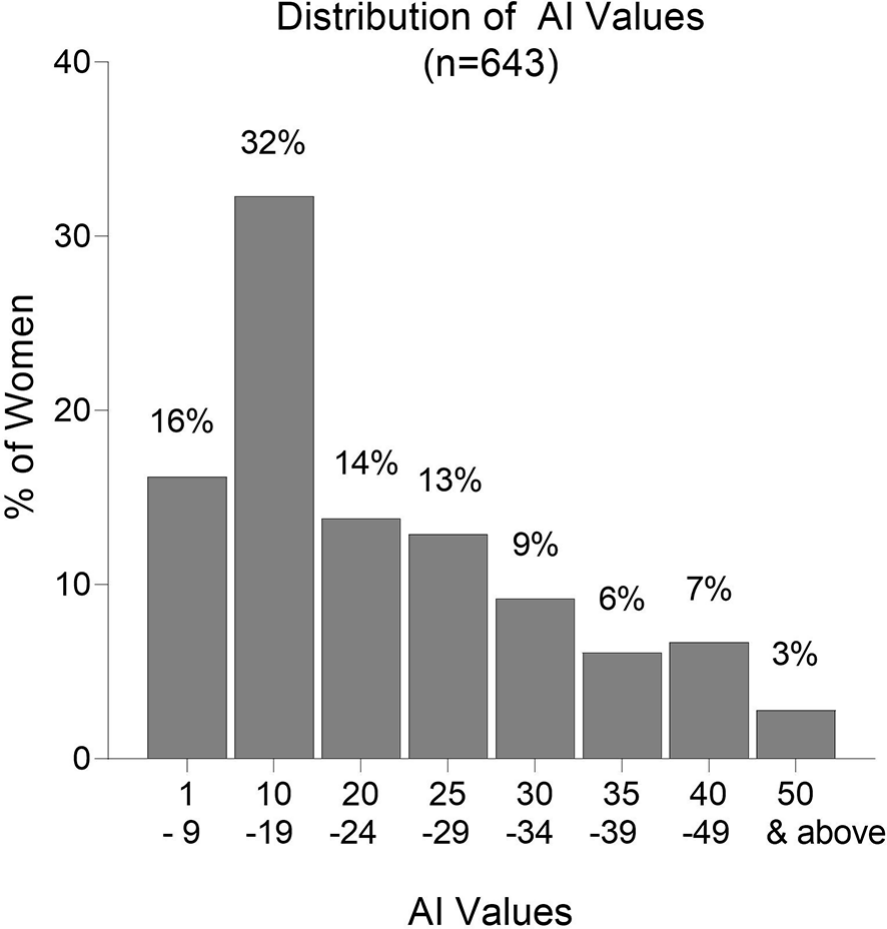
Frequency distribution of Avidity Indices (AI). Numbers above each column are the percentage of samples in the AI category. n = 643 women.

Overall, AI values were low with 48% of the women having AI <20 and 75% having AI <30 (**Figure 5**). Only 16% of women had AI ≥35; 9.5% had AI ≥40; and only 2.8% had AI ≥50. Thus, the majority of women did not produce significant amounts of high avidity Abs, and only 2.8% had AI >50, which is considered to be a high avidity response. In a high transmission area, women with AI >35 early in pregnancy were reported to have a decreased risk of PM at delivery (13). Only 16% of women in Yaoundé had this level of high avidity Abs at delivery.

### Relationship Between MFI and AI was Similar in Secundigravidae and Above

**Figure 6:**
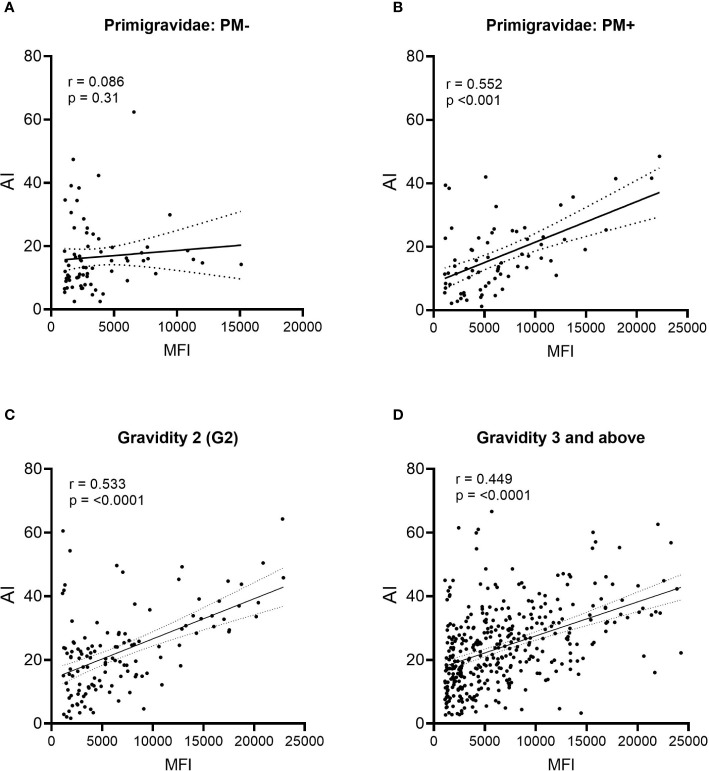
Comparison of Ab levels (MFI) and avidity (AI) using linear regression. **(A)** Placental malaria-negative (PM−) primigravidae (n = 71), **(B)** Placental malaria-positive (PM+) primigravidae (n = 70); **(C)** all secundigravidae (G2) (n = 127); and **(D)** all women gravidity 3 (G3) and above (n = 373). Linear regression lines with 95% confidence bands are shown. P values indicate if the line is significantly different from zero.

During affinity maturation, B cell clonal expansion and selection occur, resulting in an increase in both Ab levels (MFI) and avidity (AI). To help determine when affinity maturation began, the relationship between MFI and AI was assessed (**Figure 6**). No relationship existed between MFI and AI in PM− primigravidae, indicating that Abs were produced, but significant clonal selection had not occurred (*p* = 0.31, i.e., regression line was not significantly different from zero) (**Figure 6A**). A moderate association (r = 0.552), however, was detected in PM+ primigravidae, suggesting that affinity maturation was occurring at term in some women (**Figure 6B**). A modest association was also seen in secundigravidae (G2) (**Figure 6C**), as well as in women gravidae G3 and above (n=373 women) (**Figure 6D**). Even among grand multi-gravid women (G6+), the association between MFI and AI still remained modest (r = 0.508). These data suggest that affinity maturation was occurring in some primigravidae who had PM at delivery, as well as in some women with prior pregnancies.

### High Avidity Abs for Were Directed Mainly to DBL5

**Figure 7:**
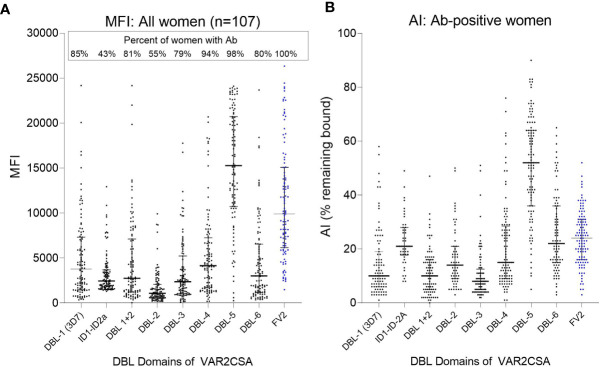
Antibody MFI and AI to different regions of VAR2CSA. Plasma samples from 107 women were screened for Ab avidity against DBL1 (3D7 line), the FCR3 lines of ID1-ID2a, the DBL2 through DBL6, and FV2. **(A)** MFI for the 107 samples. Horizonal lines represent medians and IQR. **(B)** AI for women who had Ab against each of the domains (sample size: DBL1 n = 91; ID1-ID2a n = 46; DBL1+2 n = 87; DBL2 n= 59; DBL3 n = 84; DBL4 n = 101; DBL5 n = 105; DBL6 n = 86; FV2 n = 107). Horizontal lines represent medians and IQR.

The extracellular part of VAR2CSA consists of six Duffy-binding-like domains (DBL) domains, a large intradomain (ID2) and a C-terminal region predicted to be intracellular (25). A total of 107 plasma samples with MFI to FV2 ranging from 5,000 to >20,000 were screened in the Ab avidity assay against the 6 DBL domains, ID1–ID2, and FV2 (**Figure 7**). The range of MFI for each domain, as well as the percentage of women who were Ab+, are shown in **Figure 7A**. Overall, 55% (e.g., DBL2) to 98% (e.g., DBL5) of the women had Abs to the DBL domains, with the highest Ab levels found for DBL5. Likewise, DBL5 had the highest AI compared to the other domains, in fact, the AI for DBL5 was significantly higher than the median AI to FV2 (median (25%,75% IQR): DBL5 52 (36,64) vs. FV2 24 (16,31); *p*<0.001). AI values for the other domains were much lower than to DBL5, with AI for the N-terminal domains DBL 1 through 3 having AI <20 and the more C-terminal domains, DBL 4 and DBL6 having slightly higher AI of 20 to 25. Thus, although most women had Abs to most of the domains, some regions of FV2 support affinity maturation better than others, with the majority of high avidity Abs induced by DBL5.

### Mothers With High AI Appear to Have Higher Birthweight Babies

Since the frequency distribution of AI did not identify definable groups of women (**Figure 5**), the pregnant women were divided into two groups based on the median. Women in Group 1 had AI <20.5 and Group 2 had AI ≥20.5 (**Table 2**). Compared to Group 1, women in Group 2 were older (mean age: 26.4 vs. 24.9 years, p<0.001); had higher gravidities (median 3 vs 2, p<0.001); and higher hematocrits (mean PCV: 33.5% vs. 32.4%, *p*=0.038). Women with AI above the median appeared to have higher birthweight babies than those with AI below the median (average birthweight: 3,160 vs 3,020 g, *p*=0.006). After adjusting for age and gravidity, the difference remained statistically significant (*p*=0.045), with an average difference of 104 g. The percentage of women with PM at delivery was lower in Group than 2 than Group 1 (30% vs 39%; *p* = 0.016). However, after adjusting for age and gravidity, no significant association was found between AI and presence of PM. Likewise, no relationship between low and high AI and the distribution of placental parasitemia (*p* = 0.45) was found. Thus, these data suggest that having high avidity Abs to FV2 may be associated with increased infant birthweight, but additional factors could also influence the results.

**Table 2:** Comparison of birth outcomes and AI to FV2.

	Group 1: AI <20.5 n = 319	Group 2AI: ≥20.5 n = 324	p-value^a^
Age, years [mean ± SD]	24.9 ± 6.0	26.4 ± 5.4	<0.001
Gravidity [median, (IQR)]	2 (1, 4)	3 (2, 5)	<0.001
Maternal hematocrit, % PCV [mean ± SD]^b^	32.4 ± 6.0	33.5 ± 5.7	0.038
Maternal anaemia [%]^b^	25.6 (69/270)	18.8 (48/256)	0.077
Weeks of gestation [median, (IQR)]^c^	39.7 (37.8, 40.9)	39.9 (38.1, 40.9)	0.739
Infant birth weight, g [mean + SD]^c^	3020 ± 633	3160 ± 615	0.006
Low birth weight babies [%]^c^	16.9 (50/295)	13.2 (40/304)	0.236
Placental malaria-positive (%)	39.2 (125/319)	29.9 (97/324)	0.017

^a^Statistical tests: t-tests for age, maternal hematocrit, and infant birthweight; Kruskal-Wallis tests for gravidity and weeks of gestation; Chi-square tests of independence for maternal anemia, placental malaria, and low birthweight.

^b^Analysis was performed on subjects for whom data were available, (n = 526).

^c^Only singleton deliveries were included: weeks of gestation (n = 576), infant birthweight and percent LBW (n = 599).

## Discussion

The timing of affinity maturation resulting in production of higher affinity Abs to VAR2CSA is an important component in acquiring immunity to PM. Previous studies have shown that the Ab response to VAR2CSA is different in urban cities from that in high transmission areas. For example, in Cameroon, >90% of women living in the rural village of Ngali II, where they receive ~200 infectious mosquito bites during pregnancy, had Abs to FV2 at delivery (13, 26); whereas, in Yaoundé, only 39% of women (n=1,337) were Ab+ (8). In Yaoundé, approximately half of the women who had PM at term do not have Abs to FV2 (8, 17). Thus, infection is not always accompanied by Ab production to FV2 in an urban setting. Clearly, when pregnant women receive only a few infectious mosquito bites during a single pregnancy, the resulting humoral response is limited. Results from the current study on Ab avidity help create a picture about the natural acquisition of Abs to VAR2CSA in pregnant women residing in Yaoundé. Although the data from this study are open to interpretation, the following picture is consistent with the current data set and data from past studies.

### Antibody-Levels to FV2 Remain Low in PM−Negative Primigravidae, Suggesting That Abs to Other Malarial Antigens Aid in Parasite Clearance

Primigravidae lack Abs to FV2 prior to pregnancy. Thus, primigravidae with Abs to FV2 at delivery who were PM− must have become infected early enough in pregnancy to mount an Ab-response and clear their placental infections prior to delivery. However, since Ab levels to FV2 were low, Abs to other antigens may have helped limit parasite replication and aided in parasite clearance **(** **Figure 1** **)**. This possibility is supported by results from a study that measured Ab levels in 1,377 women living in Yaoundé, (i.e., the same initial set of plasma samples used herein) to 17 VAR2CSA-associated antigens, including FV2, DBL-1-6 of the FCR3, DBL, and 7G8 lines, IDI-ID2a (FCR3 and 3D7) and 11 non-VAR antigens associated with immunity to *P. falciparum* (AMA-1, CSP, EBA-175, LAS1, MSP1, MSP2, MSP3, MSP11, Pf41, Pf70, and RESA), and then used the data in seven statistical models to identify the combination of antigens that best correlated with absence of PM at term (18). A combination of Abs to AMA-1, MSP2, EBA-175, Pf41, and MSP11 was the best predictor of PM status. FV2 was among the top 10 individual predictors, but it did not improve the prediction when added to the combination. Likewise, in a parallel study, Abs to MSP3, EBA-175 and Pf41 were associated with reduced risk of high placental parasitemia and Abs to EBA-175 with reduced risk to premature deliveries in PM+ women (8). Thus, Abs to other malaria antigens, in conjunction with FV2, may play a significant role in improving pregnancy outcomes. Since Ab level were low (**Figure 2**) in PM− primigravidae and there was no evidence that affinity maturation had occurred (**Figures 3**, **4**), initial exposure to low levels of FV2 may be sufficient to activate short-lived plasma cells, but insufficient to induce strong clonal expansion of B cells, long-lived plasma cells, memory B cells, or affinity maturation.

### Antibody-Levels to FV2 Reached Maximal Levels in PM+ Primigravidae

The anti-VAR2CSA response was significantly stronger in primigravidae who had placental parasitemia at term (**Figure 2**), possibly due to longer exposure to higher amounts of the antigen. Interestingly, distribution and median MFI of anti-FV2 Abs were similar at term in PM+ primigravidae and all other gravidity groups regardless of placental malaria status (**Figure 2**). After the first exposure to a sufficient concentration of FV2, Ab data are consistent with the speculation that B cell clonal expansion is similar in primigravidae to that of multigravida women. Thus, in primigravidae, exposure to an adequate amount of FV2 may be sufficient to induce substantial clonal expansion, but insufficient to induce affinity maturation (**Figure 3**).

### Affinity Maturation Takes Place Primarily During the Second Pregnancy

The change in the Avidity Index between primigravidae and secundigravidae (**Figures 3**, **4**) is reminiscent of a primary and secondary Ab response. During second pregnancies (PM− women) and possibly into the third pregnancies (PM+ women), affinity maturation appeared to be occurring. At this time, women had both higher Ab levels and a higher proportion of higher avidity Abs (i.e., higher AI); however, the association between MFI and AI was only modest (r values ranging from 0.4 to 0.5) (**Figures 6B, C** **)**. In general r values >0.7 are considered to be strong associations. From the scattergrams, data show that a few secundigravidae and ≥G3 had high MFI but low AI (below the line) and a group of women with low AI and high MFI (values above the line) (**Figures 6C, D** **)**. Thus, the two processes may not always occur simultaneously. Neither the quantity nor quality of Abs to FV2 appears to increase following additional exposure to VAR2CSA-expressing IE beyond the second pregnancy. These results suggest that in low transmission areas, two pregnancies are required for women to achieve the naturally acquired humoral response to VAR2CSA. However, the ultimate response is much less robust than that obtained by women living in rural areas with high exposure to VAR2CSA during pregnancy.

### High Avidity Abs Appear to be Restricted Primarily to DBL5 in this Urban Setting

In Yaoundé, most women had detectable levels of Abs to the individual DBL domains, with the predominant Ab response to DBL5 (**Figure 7A**). That is, the median Ab level to DBL5 was 15,277 MFI; whereas, median Ab levels to the other domains were <4,000 MFI (**Figure 6**). In a high transmission village, the absence of PM at term was associated with women having high Ab levels to multiple domains and strains (lines) (17). Thus, the breadth of the Ab response to VAR2CSA is much higher in a rural, compared to the urban, site. Likewise, the median AI for DBL-5 was 52 compared to <25 for the other domains (**Figure 7B**). To our knowledge, similar data are not available on the avidity of Abs to DBL domains in women in high transmission areas. In summary, in Yaoundé the Ab response was similar to a primary Ab response when primigravidae are exposed to a new antigen and secundigravidae have a secondary exposure.

The above conclusions are based on the Ab response to the FCR3 line of *P. falciparum.* Like other malarial antigens, VAR2CSA is polymorphic with six DBL domains, each of which contains conserved and polymorphic sequences. Conserved epitopes on the molecular surface that bind Abs are most predominant in DBL5, thereby, inducing strain-transcending or cross-reactivity Abs (25). Among isolates, DBL5 domains average 86% amino acid identity, which may explain, in part, the high AIs to DBL5 (27). For a vaccine to VAR2CSA to be successful, the response must be against strain transcending epitopes. A limitation of the current study is the possibility that affinity maturation occurred to a few non-strain transcending epitopes that were not detected in this study. Likewise, this study used 3M NH_4_SCN which is a very strong chaotrope for malarial antigens (22) and may efficiently remove Abs bound to more flexible epitopes. If a less stringent chaotrope had been used, additional evidence of affinity maturation may have been detected. Overall, these results suggest that in urban cities when women become infected only a few times during pregnancy, most high avidity Abs are directed against the DBL5 domain.

In high transmission areas, Abs to VAR2CSA have been reported to be associated with increase length of pregnancy, higher birthweight babies, lower placental parasitemia, and reduced prevalence of placental malaria at delivery (6, 7, 9, 10). The influence of Abs to VAR2CSA has been more difficult to assess in urban settings, in part, because a large sample size is need to detect small effects. Using data from 1,337 women, Abs to FV2 were found to be beneficial for PM+, but not PM−, women (8). That is, in PM+ women the presence of Abs to FV2 was associated with lower placental parasitemia and higher birth weight babies compared to PM+ women without Abs to FV2. In the current study, where all women had Abs to FV2, women whose AI to FV2 were above the median delivered higher birthweight babies compared to women whose AI was below the median (**Table 2**). The comparison remained significant after adjusting for age and gravidity. Thus, there is some evident that affinity maturation has a beneficial role in a low transmission setting; however, the increase of birthweight was only 104 g and may be of minimal clinical relevance. It remains unclear if Abs to FV2 with higher AI are better at blocking or reversing the attachment of infected erythrocytes to trophoblasts or if they are merely a surrogate marker for the overall maturation of the immune response to VAR2CSA.

The Ab response to VAR2CSA has been measured in other urban settings, e.g., Blantyre, (Malawi); Lambarene, Gabon; and Manjica, Mozambique (28–35). It is difficult to make direct comparisons between these cities and Yaoundé at the time the women were recruited, since factors that influence development of immunity differ among the cities, including time of the study, assays used, sample size, and range of intermediate malaria transmission. However, commonality among the results exist. In these urban cities, Ab levels in PM+ were higher than those in PM-primigravidae (28, 32, 36), Ab levels were higher in PM+ than PM− mothers at term, but there was no difference between PM+ and PM− multigravidae, regardless of the assay used to measure Abs to Var2CSA (18, 28, 33–35, 37). Similar to Yaoundé, Abs to FV2 were not associated with maternal peripheral parasitemia or percentage of LBW babies (29, 33); however, like higher avidity Abs, Abs that mediate phagocytosis of VAR2CSA-expressing IE were associated with increased infant birthweight (29). Abs, to a non-var antigen, AMA-1, were reported to contribute to improving infant birthweight (34). Thus, the results of this study may be applicable to other urban cities. With implementation of intervention strategies, such as IPT and insecticide treated bed nets, transmission of malaria is on the decline in many places (38). Today rural environments are becoming intermediate transmission areas similar to Yaoundé. Thus, the results reported herein are of both current and future interest.

## Data Availability Statement

The raw data supporting the conclusions of this article will be made available by the authors, without undue reservation.

## Ethics Statement

The part of the study involving human subjects was reviewed and approved by the National Ethics Committee, Cameroon and IRB, Georgetown University (1994-158). The patients/participants provided their written informed consent to participate in the study. The use of archival materials used in the current study was approved by the Committee on Human Subject, University of Hawaii-Manoa (CH#21891).

## Authors Contributions

The following were contributed by the authors: sample and data collection (RL), study design (NB and DT), conduction of the experiments (KV and NB), data analysis (MM and JC), provision of critical reagents and advice (AS), and manuscript preparation (RL, AS, and DT). All authors contributed to the article and approved the submitted version.

## Funding

The study was funded by NIAID, NIH UO1AI35839 (sample, clinical information, and laboratory data collection) (DT). Laboratory studies by NIAID, 1R21AI105286 (JC and DT). KV was supported by the Fogarty International Center, Global Infectious Diseases training grant D43TW009074 (DT), and MM and JC were partially supported by U54MD007601 from the National Institute of Health (NIH).

## Conflict of Interest

The authors declare that the research was conducted in the absence of any commercial or financial relationships that could be construed as a potential conflict of interest.
